# Households’ age, country of birth, and marital status, stronger predictor variables than education in the prevalence of dental sealants, restorations, and caries among US children 5–19 years of age, NHANES 2005–2010

**DOI:** 10.1186/s12903-019-0896-0

**Published:** 2019-08-27

**Authors:** Victor Alos-Rullan

**Affiliations:** Division of Community Oral Health, School of Dental Medicine, University of Pennsylvania, Philadelphia, USA

**Keywords:** Households age, Birthplace, Marital status, NHANES, Prevalence, Sealants, Restorations, Caries

## Abstract

**Background:**

The aim of this study is to use data representative of the U.S. population to determine if households (HHs) age, birth country, and marital status, are strong predictors as HHs education for dental sealants, restorations, and caries in children 5 to 19 years of age.

**Methods:**

A cross sectional analysis was performed with oral health data from three waves of the National Health and Nutrition Examination Survey (NHANES 2005 to 2010). The sample size consisted of children 5 to 19 years of age (*n* = 9151) and households > 18 years of age (*n* = 31,034). Dependent variables included the number of children with dental sealants, restorations, and caries. HHs independent variables consisted of gender, age, race, country of birth, HHs education level, marital status, and HHs spouse education. Multivariate regression analysis models were adjusted for HHs citizenship, health insurance, family size, and children age categories.

**Results:**

The prevalence of children 5–19 years of age with dental sealants, restorations, and caries was 31.3, 43, and 15.8% respectively. The odds of children having sealants were higher among HHs with a college education or above OR 2.05 [1.54.-2.73] vs. HHs with a < 9th grade, in HHs ages 39–49 (OR 1.78 [1.41–2.24) vs. 18–29 years of age, and in HHs spouses with a college education and above OR 1.71 [1.14–2.56] vs. HHs with a < 9th grade. The odds of having at least one restored tooth were higher in children from HHs born in Mexico 1.74 [1.44–2.10] vs. US born. The highest odds for caries were among children from HHs that were never married 1.91 [1.47–2.48] vs. married HHs. In HHs with a college education the odds for caries in children were 0.31 (0.22–0.43) for college and above, and 0.78 (0.60–1.01) for some college.

**Conclusions:**

The odds of children having dental sealants were higher in HHs with a college education, however, HHs ages 30–49 provided higher odds for sealants than spouses with college education. HHs birth place increased the odds of children with restorations more than HHs education. Children from HHs that never married had higher odds of experiencing dental caries. Recognizing the impact of these HHs characteristics could augment efforts in the prevention of adverse oral health outcomes in U.S. children.

Households’ age, country of birth, and marital status, stronger predictor variables than education in the prevalence of dental sealants, restorations, and caries among US children 5–19 years of age, NHANES 2005–2010.

## Background

The preponderance of evidence attests that children’s oral health is associated with multiple biological and sociodemographic factors. Likewise, income and education are frequently associated with health status globally. Households’(HHs) sociodemographic characteristics influence on children’s oral health frequently include race, income, education, and health insurance coverage [1–4]. Maternal health and marital status, birth place, work and living environments, and social support also play a significant role in children’s access to dental care [5, 6]. Households’ income and education directly correlate with children’s access to preventive and restorative dental care and indirectly with dental caries or decay [1, 7, 5, 8]. Most studies using examination data representative of the U.S. population e.g., National Health and Nutrition Examination Survey (NHANES) frequently include HHs income and education to measure associations with children’s oral health. These studies do not simultaneously measure associations that include HHs variables such as; age, gender, citizenship, country of birth, marital status, family size, with the prevalence of dental sealants, restorations, and caries in children [9, 10]. The aim of this study is to use data representative of the U.S. population to determine if households (HHs) age, birth country, and marital status, are reliable predictors as HHs education for the prevalence of dental sealants, restorations, and caries in U.S. children 5 to 19 years of age.

## Methods

### Data source and sample

A cross sectional analysis of the NHANES (2005 to 2010) oral health data sets was conducted. Written inform consent forms are collected for specimen storage and continuing studies including use of DNA. NHANES uses a multistage probability sample of approximately 5000 persons that are representative of the US civilian, noninstitutionalized population. Health interviews are conducted in respondents’ homes, and health measurements are performed in mobile examination centers. A physician, a dentist, medical and health technicians, and dietary and health interviewers, comprise the study team at examination centers [11]. Since 2005 the NHANES Basic Screening Examination (BSE) had been conducted by health technologists and in the 2009–2010 cycle by dental hygienists. The BSE does not include radiographs.

Children 5 to 19 years of age (*n* = 9151) and households older than 18 years (*n* = 31,034) were included in the study sample. NHANES examination data sets include a row for each child or person that was examined. HHs variables such as gender, age, country of birth, education, are data attributes of children that had an oral health BSE. Discrepant associations in variables are defined as those that are not in agreement with findings from peer reviewed studies using the same NHANES data sets, NHANES reported prevalence trends of dental indicators (e.g., sealants, caries), and expected outcomes associated with key determinants of health (e.g., poverty, educational).

### Dependent variables

Dependent variables include: the presence of at least one tooth with cavitated decay (ohxdecay); at least one tooth with a dental restoration (ohxrest); and at least one tooth with one dental sealant (ohxseal) for children age 5 years and older (2005 to 2008 exams cycles). In 2009–2010 the dental BSE included children 3–19 years of age, however only ages 5–19 were included in the present study. No changes in the variables for HHs were made in the study examination years (2005–2010). Binary variables were created for the 3 dependent variables (dental sealants, restorations, and caries).

From the combined 2005–2010 data sets, 2597 persons had at least one dental sealant, 10,805 a restored tooth, and 4079 one dental caries. In children 5 to 19 years of age, the present study sample consisted of 2286 with a sealant, 3819 with restored, and 1615 with caries respectively. The present study three dependent variables had less than 10% of missing data values.

### Independent variables

In NHANES a household is defined as the first household member 18 years of age or older listed on the household member roster, who owns or rents the residence where members of the household reside [12]. The present study used the following HHs demographic variables: gender (DMDHRGND), age (DMDHRAGE), country of birth (DMDHRBRN), education level (DMDHREDU), marital status (DMDHRMAR), and HH spouse education (DMDHSEDU). Analysis of race/ethnicity of Hispanics or Latinos data is provided in two categories, “Mexican Americans” and “Other Hispanics”. New categorical variables were derived from the HHs: age in four 4 categories (18–29, 30–49, 50–59, and 60 and over); country of birth in two (US, Mexico, Else); and 3 categories for marital status (married and or partner; widow, divorced, or separated; and never married). A categorical variable for children stratified ages was created (5–9, 10–14, and 15–19 years of age).

### Covariates

Bivariate measures of associations were used to identify the following potential confounders associated with the outcome variables (dental sealants, restorations, caries) and predictors HHs demographic variables: a) Income two categories, above and below 100% of the Federal Poverty Level; b) Citizenship two categories, citizen and non-citizen; c) Health Insurance two categories, with and without health insurance; d) Family Size three categories, 1–3, 4–6, and 7 or more family members, and children age categories.

### Statistical analysis

Stata version 12.1 (Texas, USA) was used for analysis. The data set was set up with survey (svy) commands to account for the complex survey design of NHANES data when determining variance estimates. A variable using the proper weight formula was created following NHANES guidelines (gen wtmec6yr = 1/3*wtmec2yr). An age standardization weight variable was created using NHANES methods and US Census 2000 population data.

Data sets containing demographic, dental examination, and health insurance variables were merged for each cycle (i.e., 2005–2006, 2007–2008, and 2009–2010). The merged files were then appended to a single data file. The sum of persons with dental sealants, restorations, and caries, in each individual exam cycle was verified for accuracy with the total sum found in the appended data file.

Bivariate analysis provided the unweighted sample size and weighted percentage for dependent, independent, and covariate variables of both children and HHs. This was stratified by the following race categories: Mexican American, Other Hispanic, Non-Hispanic White, Black, and Other Race including multiracial. Bivariate analysis was used to calculate the prevalence of children (ages 5–19) with sealants, restorations, and caries by each of the HHs demographic variables. The same method was used to determine associations between HHs predictor variables and covariates. In similar associations between HHs characteristics and children clinical outcomes, the smallest proportion of preventive interventions, e.g. sealants, would be expected to result in a higher proportion of restored teeth and caries – HHs age 18–29. A conflicting association in a smaller proportion of sealants would entail lower proportions of restored teeth and caries- male HHs vs females (Table 3).

Logistic regression was used to calculate the odds of children having dental sealants, restorations, and caries, by each of the HHs predictor variables (i.e., gender, age, country of birth, education, marital status, and spouse education). For each of the three dental dependent variables an unadjusted model was followed by adjustments for HHs income and insurance coverage (Model 1), and Model 1 plus HHs citizenship and family size (Model 2) not shown. After removing income the odds ratios did not change 10% or more and the variable was deleted from the adjusted models. Children age categories were added in the final adjusted model.

The Stata variance inflation factor (vif) command after the regression was used to check for multicollinearity. Covariate variables for race, income, health insurance, children’s age, and citizenship were tested for interaction with predictor variables and dependent variables.

## Results

From the study sample of children 5–19 years of age (*n* = 9151), 51.1% were males, 32, 33.91 and 34.09%, were 5–9, 10–14, 15–19 years of age respectively, and 20.5% lived under 100% of the Federal Poverty Level (FPL) (Table 1). The study HHs sample (*n* = 31, 034) included 54.7% males, 47.9% in the age range 30–49, 26.6% with a college or above education, 14.9% living below 100% of the FPL, 11.4% never married, 56.4% had a family size of 1–3 members, 82.8% had health insurance, and 92.2% were US citizens. Mexican Americans and other Hispanics HHs had the highest proportion of persons living under 100% of the FPL, with less than a 9th grade education, no health insurance and a non-citizen status. HHs from Other Race/Multiracial had the highest percentage of being married or living with a partner, Mexican Americans, Non-Hispanic White, and Other Hispanic ranked 2nd, 3rd, and 4th (Table 2).

**Table 1:** Selected Characteristics of Children 5–19 years of age, NHANES, 2005–2010 (*n* = 9151).

		Mexican American	Other Hispanic	Non-Hispanic White	Non-Hispanic Black	Other Race/Multi-Racial	Total	*P* value
N (%)^a^		2668	787	2701	2479	516	9151	
Sex								0.4658
Male		1345 (51.1)	397 (48.9)	1398 (51.9)	1259 (50.0)	256 (49.0)	4655 (51.1)	
Female		1323 (48.9)	390 (51.1)	1303 (48.1)	1220 (50.0)	260 (51.0)	4496 (48.9)	
Age								0.0084
5–9		921 (36.66)	281 (33.68)	914 (30.66)	756 (31.27)	188 (34.42)	3060 (32.00)	
10–14		896 (33.99)	259 (32.55)	876 (33.71)	832 (33.76)	174 (36.83)	3037 (33.91)	
15–19		851 (29.35)	247 (33.77)	911 (35.63)	891 (34.97)	154 (28.75)	3054 (34.09)	
Education (age 6–19)								0.0376
Never Attended		209 (9.22)	63 (7.60)	215 (7.95)	175 (7.80)	50 (8.90)	712 (8.14)	
1st – 8th		1483 (62.0)	439 (59.2)	1437 (57.2)	1318 (57.3)	288 (63.2)	4965 (58.3)	
9th – 12th		693 (26.6)	203 (29.5)	731 (30.1)	712 (30.1)	122 (23.2)	2461 (29.4)	
> HS		60 (2.40)	24 (3.70)	103 (4.20)	111 (4.81)	21 (4.72)	319 (4.05)	
Poverty								< 0.0001
< 100% FPL		947 (36.0)	267 (32.8)	479 (12.8)	827 (33.6)	127 (19.5)	2647 (20.5)	
> 100% FPL		1711 (64.0)	520 (67.2)	2205 (87.2)	1642 (66.4)	388 (80.5)	6466 (79.5)	
Dental Indicators	Ages							
Sealant	6–9	170 (24.1)	49 (21.7)	182 (27.8)	114 (20.0)	34 (26.7)	549 (25.7)	0.1051
	13–15	148 (34.4)	59 (38.7)	219 (44.7)	112 (26.4)	40 (49.7)	578 (40.9)	< 0.0001
Restoration	6–9	383 (54.0)	87 (39.7)	248 (36.7)	195 (34.2)	47 (30.6)	960 (38.7)	0.0001
	13–15	248 (52.3)	66 (46.1)	236 (45.0)	197 (43.1)	53 (57.9)	800 (46.6)	0.0489
Caries	6–9	191 (27.1)	43 (19.5)	121 (16.5)	152 (26.5)	32 (18.0)	539 (19.8)	0.0008
	13–15	84 (18.0)	16 (13.2)	49 (7.92)	86 (20.1)	10 (9.17)	245 (11.2)	< 0.0001
Sealant	5–19	640 (26.7)	212 (28.1)	822 (34.4)	458 (21.0)	154 (37.5)	2286 (31.3)	< 0.0001
Restoration	5–19	1308 (51.9)	314 (43.3)	1070 (41.6)	921 (40.4)	206 (43.7)	3819 (43.0)	0.0011
Caries	5–19	546 (22.0)	120 (16.2)	362 (12.7)	503 (22.5)	84 (16.4)	1615 (15.8)	< 0.0001

Federal Poverty Level (FPL)

100% of Federal Poverty Level for 1 person ($10,830), 2 persons ($14,570), 4 persons ($22,050)

^a^Unweighted sample size and weighted percentage of subjects in each strata of subject characteristic

**Table 2:** Selected Characteristics of Households > 18 years of age, NHANES, 2005–2010

	Mexican American	Other Hispanic	Non-Hispanic White	Non-Hispanic Black	Other Race Including Multi-Racial	Total	*P* value
N (%)a	7388	2683	12,463	6878	1622	31,034	
Sex							< 0.0001
Male	4225 (59.0)	1236 (48.6)	6788 (56.7)	2824 (41.9)	833 (56.2)	15,906 (54.7)	
Female	3163 (41.0)	1447 (51.4)	5675 (43.3)	4054 (58.1)	789 (43.8)	15,128 (45.3)	
Age							< 0.0001
18–29 yrs.	1634 (22.7)	461 (17.4)	1777 (12.7)	1320 (19.4)	282 (15.7)	5474 (14.9)	
30–49 yrs.	3985 (55.4)	1416 (57.1)	5517 (45.7)	3186 (46.9)	914 (54.5)	15,018 (47.9)	
50–59 yrs.	838 (12.4)	327 (12.5)	1740 (18.4)	1022 (17.3)	221 (17.7)	4148 (17.3)	
60+ yrs.	931 (9.50)	478 (13.0)	3429 (23.2)	1350 (16.3)	203 (12.2)	6391 (19.8)	
Education							< 0.0001
< 9th	2303 (31.6)	481 (17.8)	534 (2.85)	288 (3.90)	124 (6.60)	3730 (6.80)	
9-11th	1580 (21.8)	534 (19.9)	1442 (9.54)	1518 (22.0)	160 (7.85)	5234 (12.6)	
HS/GED	1469 (20.4)	552 (21.5)	3246 (25.5)	1703 (26.2)	317 (17.4)	7287 (24.3)	
Some College	1209 (18.2)	652 (26.7)	3714 (31.4)	2171 (33.3)	390 (25.1)	8136 (30.0)	
College +	507 (8.04)	355 (14.1)	3223 (30.8)	994 (15.0)	565 (43.0)	5644 (26.6)	
Poverty							< 0.0001
< 100% FPL	2518 (32.7)	787 (26.9)	1958 (9.50)	1880 (25.0)	382 (15.2)	7525 (14.9)	
> 100% FPL	4844 (67.3)	1896 (73.1)	10,459 (90.5)	4977 (75.0)	1236 (84.8)	22,953 (85.1)	
Marital Status							< 0.0001
Married/Living partner	5311 (74.1)	1727 (68.5)	8646 (72.9)	3114 (47.7)	1137 (77.3)	19,935 (70.1)	
Widowed/Divorced/ Separated	1132 (15.4)	506 (17.9)	2551 (18.5)	1724 (25.5)	234 (12.0)	6147 (18.6)	
Never married	664 (10.5)	355 (13.5)	1050 (8.62)	1762 (26.7)	190 (10.7)	4021 (11.4)	
Family Size							< 0.0001
1–3	2100 (32.1)	1080 (41.7)	7435 (62.5)	3443 (54.1)	702 (48.5)	14,760 (56.4)	
4–6	3987 (52.3)	1276 (47.9)	4598 (35.0)	2970 (40.5)	778 (46.3)	13,609 (38.9)	
7 +	1301 (15.6)	327 (10.4)	430 (2.50)	465 (5.40)	142 (5.20)	2665 (4.70)	

Federal Poverty Level (FPL)

100% of Federal Poverty Level for 1 person ($10,830), 2 persons ($14,570), 4 persons ($22,050)

^a^Unweighted sample size and weighted percentage of subjects in each strata of subject characteristic

In bivariate analysis, sealants were more prevalent among children from HHs with spouses with a college or above education (40.2%), HHs with a college or above education (39.9%), non-Hispanic White HHs (34.4%), HHs ages 50–59 (33.9%), and ages 30–49 (32.9%), and HHs with insurance (32.6%). Children from non-citizen HHs had the lowest sealant prevalence (20%). Children from HHs of Mexican-American ancestry and birth place had the highest prevalence of restored teeth (51.9%) and (50.3%) respectively. A college education or above for both the HHs and their spouses resulted in the lowest prevalence of restored teeth (32.9%) and (34%) respectively in children. Children’s caries prevalence was higher among non-citizens and uninsured HHs (30.1%) and (27.9%), and lowest in children from HHs and spouses with college of above education (6.9%) (Table 3).

**Table 3:** Children’s Sealant, Restoration, and Caries prevalence by Household Characteristics, NHANES, 2005–2010

Households’ Characteristics	Dental Outcomes in Children 5–19 years of age (%) ^a^					
	Sealants	*P* value	Restorations	*P* value	Caries	*P* value
Sex		0.1028		0.2153		0.0174
Male	30.1%		42.2%		14.5%	
Female	32.5%		43.9%		17.3%	
Age, years		< 0.0001		0.1310		0.0158
18–29	20.6%		46.3%		20.6%	
30–49	32.9%		42.0%		15.2%	
50–59	33.9%		45.0%		14.6%	
60+	24.5%		46.3%		19.2%	
Birth Country		0.0815		0.0102		0.0003
US	32.0%		42.5%		15.3%	
Mexico	25.8%		50.3%		23.0%	
Else	31.9%		40.9%		14.6%	
Education		< 0.0001		< 0.0001		< 0.0001
< 9th	22.0%		49.3%		24.4%	
9-11th	24.1%		49.3%		22.1%	
High School	29.2%		44.2%		19.6%	
Some College	31.8%		45.0%		15.5%	
College	39.9%		34.0%		6.8%	
Race		< 0.0001		0.0011		< 0.0001
Mexican American	26.7%		51.9%		22.0%	
Other Hispanic	28.1%		43.3%		16.2%	
Non-Hispanic White	34.4%		41.6%		12.7%	
Non-Hispanic Black	21.0%		40.4%		22.5%	
Other Race / Multi-Racial	37.5%		43.7%		16.4%	
Poverty		< 0.0001		0.0067		< 0.0001
> 100% FPL	33.2%		42.0%		13.7%	
< 100% FPL	24.2%		47.4%		24.3%	
Citizen		0.0002		0.1019		< 0.0001
Yes	31.9%		43.3%		15.1%	
No	20.0%		38.0%		30.1%	
Insurance		< 0.0001		0.9431		< 0.0001
Yes	32.6%		43.0%		14.2%	
No	21.8%		43.2%		27.9%	
Education Spouse		< 0.0001		< 0.0001		< 0.0001
< 9th	24.7%		49.7%		20.7%	
9-11th	28.7%		48.2%		20.7%	
HS/GED	27.2%		42.4%		14.3%	
Some College	32.4%		42.4%		15.6%	
College +	40.2%		32.9%		6.9%	
Marital Status		0.0573		0.0100		< 0.0001
Married/partner	32.2%		41.3%		14.2%	
Widow/divorced/separated	31.3%		45.9%		18.1%	
Never married	25.7%		45.9%		22.0%	
Family Size		0.0094		0.0115		0.0057
1–3	31.6%		46.2%		15.2%	
4–6	32.0%		41.3%		15.3%	
7+	24.7%		47.0%		22.2%	

Federal Poverty Level (FPL)

100% of Federal Poverty Level for 1 person ($10,830), 2 persons ($14,570), 4 persons ($22,050)

^a^Unweighted sample size and weighted percentage of subjects in each strata of subject characteristic

After adjusting for HHs health insurance, citizenship, family size, and children’s age categories, the odds of children having sealants were higher among HHs with a college education or above OR 2.05 [1.54.-2.73] vs. HHs with a < 9th grade, in HHs ages 39–49 (OR 1.78 [1.41–2.24) vs. 18–29 years of age, and in HHs spouses with a college education and above OR 1.71 [1.14–2.56] vs. HHs with a < 9th grade. Lowest odds for sealant were among children from HHs of non-Hispanic Black ancestry 0.60 [0.48–0.75]. The odds of having restored teeth were higher in children of HHs born in Mexico 1.74 [1.44–2.10], never married HHs 1.21 [1.02–1.43] and those widowed/divorced/separated 1.18 [1.01–1.39]. Lowest in HHs with college or above education 0.46 [0.33–0.63]. The highest odd for caries was among children from HHs that were never married 1.91 [1.47–2.48]. Children from female HHs had an OR of 1.26 (1.06–1.50) for caries when compared to male HHs. Children living in HHs with a college education 0.31 [0.22–0.43] had the lowest odds for caries (Table 4).

**Table 4:** Children (5–19 years) Odds Ratio sealants, restorations, caries and Household Characteristics, NHANES, 2005–2010

Households Characteristics	Adjusted^a^ OR (95% CI) Adjusted^a^ OR (95% CI) Adjusted^a^ OR (95% CI)		
	Sealants	Restorations	Caries
Sex			
Male	1	1	1
Female	1.11 (0.97–1.26)	1.07 (0.95–1.20)	1.26 (1.06–1.50) [3]
Age, years			
18–29	1	1	1
30–49	1.78 (1.41–2.24) [1]	0.79 (0.66–0.95) [3]	0.75 (0.61–0.93) [3]
50–59	1.65 (1.22–2.23) [2]	0.77 (0.62–0.97) [3]	0.83 (0.63–1.10)
60+	1.12 (0.76–1.66)	0.87 (0.62–1.21)	1.01 (0.57–1.79) [3]
Race/Ethnicity			
Mexican American	1	1	1
Other Hispanic	0.99 (0.72–1.37)	0.65 (0.50–0.85) [2]	0.76 (0.59–0.98) [3]
Non-Hispanic White	1.18 (1.00–1.40)	0.55 (0.45–0.67) [1]	0.69 (0.55–0.86) [2]
Non-Hispanic Black	0.60 (0.48–0.75) [1]	0.53 (0.45–0.63) [1]	1.40 (1.15–1.71) [2]
Other / Multiracial	1.50 (1.12–2.00) [3]	0.66 (0.50–0.87) [2]	0.82 (0.61–1.12)
Birth Country			
US	1	1	1
Mexico	0.96 (0.77–1.18)	1.74 (1.44–2.10) [1]	1.02 (0.80–1.32)
Else	1.12 (0.86–1.49)	1.04 (0.85–1.28)	0.74 (0.57–0.96) [3]
Education			
< 9th	1	1	1
9-11th	1.03 (0.78–1.37)	0.92 (0.72–1.18)	1.09 (0.87–1.36)
High School	1.31 (1.03–1.68) [3]	0.72 (0.59–0.92) [3]	0.98 (0.81–1.20)
Some College	1.42 (1.12–1.82) [3]	0.73 (0.57–0.92) [3]	0.78 (0.60–1.01) [3]
College or above	2.05 (1.54–2.73) [1]	0.46 (0.33–0.63) [1]	0.31 (0.22–0.43) [1]
Marital Status			
Married/partner	1	1	1
Widow/divorced/separated	0.90 (0.75–1.09)	1.18 (1.01–1.39) [3]	1.50 (1.24–1.80) [1]
Never married	0.69 (0.53–0.92) [3]	1.21 (1.02–1.43) [3]	1.91 (1.47–2.48) [1]
Education Spouse			
< 9th	1	1	1
9-11th	1.07 (0.64–1.66)	0.89 (0.62–1.28)	1.32 (0.92–1.88)
HS/GED	0.92 (0.59–1.42)	0.67 (0.47–0.94) [3]	0.91 (0.65–1.28)
Some College	1.21 (0.81–1.80)	0.68 (0.50–0.93) [3]	1.01 (0.70–1.46)
College or above	1.71 (1.14–2.56) [3]	0.46 (0.33–0.64) [1]	0.41 (0.29–0.58) [1]

^1^(*P* < 0.001) ^a^Adjusted for household reference person insurance, citizenship, family size, children age

^2^(*P* < 0.005) Federal Poverty Level (FPL), High School (HS), General Educational Development (GED)

^3^(*P* < 0.05) Federal Poverty Level (FPL) 100% of Federal Poverty Level for 1 person ($10,830), 2 persons ($14,570), 4 persons ($22,050)

## Discussion

Results from this study indicate that after controlling for health insurance, citizenship, family size, and children age categories, having HHs with ages 30–49 could significantly have higher odds of a child receiving dental sealants than HHs spouses with a college education or above. HHs birth place increased the odds of children’s experiencing dental restorations more than HHs with college education. HHs with a marital status “Never Married” provided the highest odds for dental caries in children.

To the best of the author’s knowledge, this study is the first to use recent NHANES data to measure the associations between HHs gender, age categories, marital status, country of birth, and the prevalence of children’s dental sealants, restorations, and caries. The findings in this study agree with other research that reports disparities in Mexican-American and Black or African-American children’s prevalence of caries and dental sealants [13–15]. Similar to the present study researchers have found positive associations between children prevalence of dental sealants and parents with higher educational attainment and having dental insurance [16–18]. Bivariate analysis in the present study confirms that the prevalence of children’ dental sealants, restorations, and caries was higher in homes where females HHs (owns or rents house) when compared to male HHs. After adjusting for covariates, female HHs only significantly increased the odds of children having caries. Although not significant, children from female HHs had slightly higher odds for sealants and restorations. Higher health literacy among female HHs and a better understanding of the value of prevention (sealants) may explain the present study findings on children’s dental sealants. After adjusting for race, education, age, and number of children, female oral health literacy has been associated with better reported oral health status [19].

In the present study the highest odds of having children with dental caries were linked to HHs that never married followed by HHs widows/separated/divorced (Table 4). The high risk of caries in children has been associated with single-parent households [20–22]. Authors using NHANES data reported that even though disparities in caries prevalence may be decreasing across racial and ethnic groups, only non-Hispanic Black children 15 year of age experienced a significant decrease in caries experience [23]. These findings differ from the present study where in Non-Hispanic Black HHs the odds of children’s dental caries were 40% higher when compared to children’s odds from “Mexican American” HHs. In a systematic review on parental influences on the development of caries in children aged 0–6 years, the age of the mother had no clear effect on the risk of caries in children [24]. Similarly the present study did not find significant associations between HHs ages 50–59 and the odds of children having caries. However both of these studies findings differ from a longitudinal study where caregivers age was associated with new non-cavitated caries lesions in children 2–5 years of age [25].

The present study was not able to measure the influence of HHs oral health literacy on children’s dental indicators. Caregivers’ oral health literacy has been associated with children oral health status [26, 27]. NHANES data on fluoride products as dietary supplements are not linked to subjects participating in the oral health BSE and the confounding effects of fluoride on the prevalence of restorations and caries could not be measured in the present study. Underreporting of caries or tooth decay may result in NHANES studies since the BSS does not include radiographs that can detect interproximal lesions. In the United States (US), poverty is measured by the Federal Poverty Level (FPL). During 2010, families living at the US 100% of Federal Poverty Level were consider poor if their annual income was $10,830 for one person, $14,570 for two persons, and $22,050 for 4 persons. In 2014, US median income was higher among those aged 45 to 54 and married couples, and more females and persons without a high school diploma lived in poverty [28]. Worldwide poverty is measured differently from the US. Age, marital status, and education effects on poverty might not be similar to the US. Results from this study could possibly not be valid in other countries.

## Conclusions

HHs age, marital status, and gender, could contribute to a better understanding of the root causes of oral health disparities in children. Knowledge of the aforementioned HHs characteristics impact on children’s access to dental services could help providers identify families with children who are at a greater risk for caries or not benefiting from preventive dental services. Tailored outreach, case management, and patient education interventions could help families and providers to work on strategies to improve children’s oral health.

## Data Availability

For this study I used publicly available data sets from three waves (2005 to 2010) of the National Health and Nutrition Examination Survey (NHANES) available at: https://wwwn.cdc.gov/nchs/nhanes/Default.aspxPlease note that from the link above one must export SAS files from each of the years (2005–2006), (2007–2008), and (2009–2010) and different data files with examination, questionnaire, and sociodemographic characteristics. All of these then need to be appended into a single data file. I can provide the STATA do files and the single consolidated data file for other to replicate my tables and data.

## Abbreviations

BSE: Basic Screening Examination
DMDHRAGE: Age
DMDHRBRN: Country of birth
DMDHREDU: Education level
DMDHRGND: Gender
DMDHRMAR: Marital status
DMDHSEDU: Spouse education
FPL: Federal Poverty Level
HHs: Households
NHANES: National Health and Nutrition Examination Survey
ohxdecay: Cavitated decay
ohxrest: Dental restoration
ohxseal: Dental sealant
svy: STATA survey command
USA: United States of America
vif: STATA variance inflation factor command
